# Should the basophil activation test be the gold standard in the diagnosis of food allergies?

**DOI:** 10.1186/1753-6561-9-S1-A61

**Published:** 2015-01-14

**Authors:** E Khaleva, N Bychkova, G Novic

**Affiliations:** 1 Sankt-Petersburg State Medical Pediatric University, St Petersburg, Russia; 2 Nikiforov Russian Center of Emergency and Radiation Medicine, St Petersburg, Russia

## Background

The basophil activation test (BAT) is an in vitro test which allows to identify children with food allergies at the sensitization stage and clinical manifestations of atopic dermatitis\\eczema (AD). The aim of our study was to observe the BAT in children with food allergies, optimize the diagnosis compared with other tests, and select an elimination diet. Early detection of sensitization and elimination of causative allergens can help prevent the progression of the disease into bronchial asthma in such children.

## Methods

We investigated 89 children from 3 months to 12 years with FA experience and AD symptoms in varying severity. We used the BAT by flow cytometry (CD203C +), specific IgE, reaction of mast cell degranulation (RMCD) in rats, and the prick skin test.

## Results

The level of spontaneous activation of basophils (SAB) it means basic expression of basophiles, depended on the severity of AD (p <0.05) and did not depend on the period of the disease (recurrence or remission).The level of SAB, allergen-induced basophil activation was significantly higher in the polyvalent sensitization group than in the monovalent sensitization group.(p <0.05). We found positive basophil activation in 25% of specific IgE negativity, in 30% of RMCD negativity.Use of selection elimination diet based on the results achieved using BAT allowed us to achieve sustained remission in 80% of the patients.

## Conclusions

BAT is a highly sensitive and accurate diagnostic method of sensitization in children with FAs which are manifested in the form of AD. Although accurate, it should not be used alone. Instead, it best used with other complementary tests for the clearest representation of each condition. Thus, the BAT should be recommended to help prescribe elimination diets for patients with FA.

